# Clinical Effect of Laminectomy with Lateral Mass Screw Fixation in Treating Cervical Schwannoma: A Retrospective Study

**DOI:** 10.1155/2022/8512374

**Published:** 2022-04-29

**Authors:** Xiaohui Guo, Sidong Yang, Zhaohui Li, Dalong Yang, Wenyuan Ding

**Affiliations:** ^1^Department of Spine Surgery, The Third Hospital of Hebei Medical University, Shijiazhuang 050051, China; ^2^Department of Spine Surgery, The Second Hospital of Tangshan, Tangshan 063000, China

## Abstract

**Background:**

The objective of this study was to evaluate the clinical effectiveness and safety of laminectomy combined with lateral mass screw fixation in treating cervical intradural extramedullary schwannoma.

**Methods:**

We retrospectively collected and analyzed medical records of 38 patients who underwent resection of cervical intraspinal schwannoma between January 2012 and April 2019. Based on different surgical procedures, two groups were divided among all participants: laminectomy-only (*n* = 21) and laminectomy with instrumented fixation (*n* = 17); the minimum follow-up time was 1 year. The visual analogue scale (VAS) score and neck disability index (NDI) were utilized for pain assessment; the Japanese Orthopedic Association (JOA) score was carried out for the assessment of neurological impairment. Radiographic changes of Cobb angle were compared before and after the surgery.

**Results:**

Consequently, demographics were well matched in both groups, without any statistical difference (*P* > 0.05). Compared with preoperation, both surgical procedures significantly improved VAS, NDI, and JOA scores (*P* < 0.001), but no differences between them (*P* > 0.05). In terms of postoperative spinal instability/deformity, laminectomy-only caused more events than instrumented fixation, which is statistically significant (*P* < 0.001).

**Conclusions:**

In summary, laminectomy with lateral mass screw fixation is an effective and safe approach to treat cervical intraspinal schwannoma, which is likely to be a better choice than the laminectomy-only approach.

## 1. Introduction

Schwannomatosis is a distinct syndrome with the characterization of multiple peripheral nerve schwannomas, familial or sporadic [1]. Both neurofibromas and schwannomas are composed of neoplastic Schwann cells [2–4]. Spinal schwannomas are benign tumors, and most of them are extramedullary intradural [5–8]. However, there are anatomopathological differences between neurofibromas and schwannomas. Neurofibromas are areas of increased thickness of the nerve, often dumbbell-shaped and sited next to the intervertebral foramina. Schwannomas are well demarcated, encapsulated, typically round, and attached to the nerve roots [9].

Reportedly, 24% of all nerve sheath tumors in adults are schwannomas, which are the most frequent extramedullary, intradural spinal tumors [9]; intradural Schwannoma may rank up to a percentage of 83.67%. To our knowledge, the main symptoms caused by intradural extramedullary schwannoma are radiculopathy and neurogenic claudication, due to spinal cord compression with the growth of tumor [10, 11]. What is more, it usually causes motor loss and worsening sensory, as well as back pain spreading out from the tumor level [12, 13]. To date, surgical resection of the tumors by posterior laminectomy is still the first choice for the treatment of symptomatic intraspinal schwannomas [14]. However, the laminectomy-only approach might lead to the acceleration of spinal degeneration and progress to spinal instability and even form spinal deformity, due to the destruction of posterior column structure [15, 16].

Thus, the current study was focused on the assessment of clinical effectiveness and safety of posterior laminectomy plus lateral mass screw fixation in the treatment of cervical intraspinal schwannomas by comparing with a laminectomy-only approach, based on a minimum of 1-year follow-up. Specifically, we only enrolled patients who had sustained cervical intradural extramedullary schwannomas to minimize the confounding factors.

## 2. Patients and Methods

### 2.1. Ethical Statement

This retrospective study has been approved by the local Ethical Committee of the Third Hospital of Hebei Medical University. This study was supported by all the participants with their informed consent provided. The methods that were used in this study were conducted according to related regulations and guidelines.

### 2.2. Participant Selection

**Figure 1:**
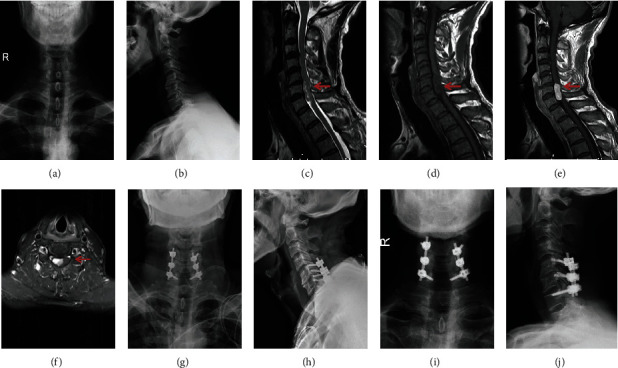
Radiological images before surgery, after surgery, and at the last follow-up. (a, b) Preoperative X-ray images; (c, d) preoperative T2- and T1-weighted MRI scan; (e, f) preoperative enhanced T1-weighted MRI scan; (g, h) postoperative X-ray immediately; (i, j) postoperative X-ray at the last follow-up.

In the current study, all participants were screened by medical records. The identified patients had experienced resection surgery to remove cervical intradural extramedullary schwannomas by total laminectomy, with or without instrumented fixation (Figure 1). All cases in this study received surgeries for the first time after being diagnosed with cervical spinal schwannomas; they did not have a previous history of the same diseases. No other tumors were found concurrent at the moment of surgery. During the period of follow-ups, diseases that were newly found and can change spinal stability have been excluded, such as severe osteoporosis, ankylosing spondylitis, and spine trauma. Patients undergoing reoperations were also excluded if performed due to recurrence. Our patients were usually followed up after surgery regularly, with postoperative 3rd month and 12th month and thereafter.

### 2.3. Clinical Assessment

Clinical assessment was conducted, and radiological changes were recorded preoperatively and postoperative 3rd month and 12th month and the last follow-up (1 year or longer). The visual analogue scale (VAS) score and neck disability index (NDI) were utilized for pain assessment; the Japanese Orthopedic Association (JOA, 17 points) score was carried out for the assessment of neurological impairment. Radiographic changes of Cobb angle were compared before and after the surgery. In addition, postoperative complications were recorded and compared. Furthermore, an analysis of patient satisfaction was performed as before [17, 18], according to the questionnaire including three levels of satisfaction—very satisfied, satisfied, and dissatisfied.

### 2.4. Statistical Analysis

Statistics was done with SPSS for Windows 18.0 (IBM SPSS Inc., Chicago, IL). Measurement data were presented as mean ± SD (standard deviation). The comparisons regarding VAS score, NDI, and JOA score between presurgery and postsurgery were conducted by analysis of variance (ANOVA), with SNK-q tests as post hoc tests. Student's *t*-tests were used to compare demographic data and surgical parameters. A chi-square test was used to compare categorical data between groups. Statistical significance was identified when *P* < 0.05.

## 3. Results

### 3.1. Demographics and Baseline Data

We retrospectively collected and analyzed medical records of 38 patients who underwent resection of cervical intraspinal schwannoma between January 2012 and April 2019. Based on different surgical procedures, two groups were divided among all participants: laminectomy-only (*n* = 21) and laminectomy with instrumented fixation (*n* = 17); the minimum follow-up time was 1 year. As shown in Table 1, the age of the laminectomy-only group was 49.4 years (19–72), while that of the lateral mass screw group was 47.8 years (17–75). There were 12 males and 9 females in the laminectomy-only group and 11 males and 6 females in the lateral mass screw group. Preoperative symptom duration was 7.3 ± 4.4 months and 6.9 ± 4.8 months in the laminectomy-only group and the lateral mass screw group, respectively. The follow-up period was 40.6 ± 21.5 months and 43.1 ± 19.8 months in the laminectomy-only group and the lateral mass screw group, respectively. There is no significant difference between these two follow-up periods (*P* > 0.05). Comparisons between the two groups above did not show any differences in terms of age, sex percentage, duration of symptom, follow-up, blood loss, and hospital stay (all *P* > 0.05). Blood transfusion was not compared between the two groups due to a lack of sufficient data.

**Figure 2:**
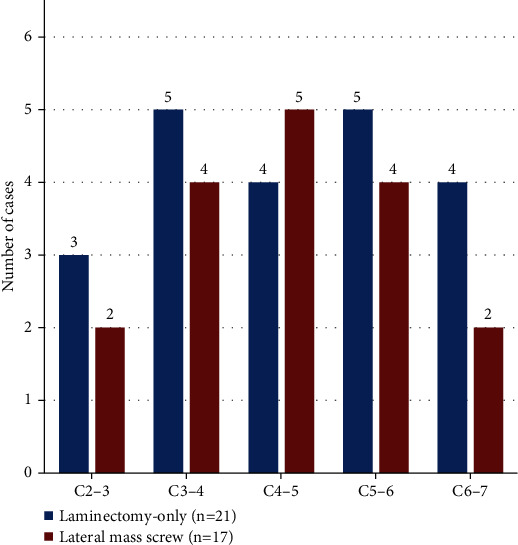
Distribution of cervical intraspinal schwannoma preoperatively.

However, the lateral mass screw group underwent longer surgical time and higher medical expenses in comparison with the laminectomy-only group (*P* < 0.05). The segmental distribution of cervical intraspinal schwannoma is shown in Figure 2. It revealed a similar distribution between the laminectomy-only group and the lateral mass screw group.

### 3.2. VAS Score and NDI

As exhibited in Table 2, the preoperative VAS score was 5.18 ± 2.01 and at the last follow-up was 1.02 ± 0.25 in the laminectomy-only group. The preoperative VAS score was 5.21 ± 2.13 and at the last follow-up 1.05 ± 0.22 in the lateral mass screw group. As shown in Table 3, preoperative NDI was 24.5 ± 13.8 and at the last follow-up 4.6 ± 2.5 in the laminectomy-only group, while they were 23.8 ± 14.6 and 4.3 ± 2.4, respectively, in the lateral mass screw group. Statistical analysis showed that VAS score and NDI have significantly improved in the laminectomy-only and lateral mass screw groups, compared with the preoperative ones (all *P* < 0.001). And yet, VAS score or NDI comparisons did not indicate any significant differences between the two groups above.

### 3.3. JOA Score

As Table 4 tells, in the laminectomy-only group, the preoperative JOA score was 7.1 ± 3.5 and postsurgery was 13.0 ± 2.6 at the last follow-up; in the lateral mass screw group, preoperative JOA score was 7.3 ± 4.1 and postsurgery 12.8 ± 2.5 at the last follow-up. Statistically, the within-group differences were significant regarding the JOA score between the postoperative and the preoperative ones (*P* < 0.001). No differences were found between the laminectomy-only and lateral mass screw groups, regardless of preoperation, postoperative 3rd month, postoperative one year, and the last follow-up (all *P* > 0.05).

### 3.4. Complications

Table 5 has summarized the main postoperative complications. Statistics has indicated that spinal instability, even deformity formation, only occurs in the laminectomy-only group, more than in the lateral mass screw group (*P* < 0.001). No differences were found between these two surgical procedures regarding the other complications (all *P* > 0.05).

### 3.5. Patient Satisfaction

Patient satisfaction grades are collected in Table 6; it did not show any significant difference between the laminectomy-only group and the lateral mass screw group regarding patient satisfaction grades (*χ*^2^ = 646, *P* = 0.724). Most patients were very satisfied with their surgical outcomes.

## 4. Discussion

Clinically, schwannomas are found the most common type of spinal intradural nerve sheath tumors, the second neurofibromas [19]. Because of sharing some similarities in symptoms and imaging characteristics, intradural extramedullary schwannoma and intervertebral disc diseases could be misdiagnosed to each other. Diagnosis of cervical radiculopathies due to disc herniation is straightforward with MRI or CT scan and electromyography. Schwannomas are relatively rare and often initially asymptomatic and yet can be progressive to manifest paresthesia, pain, numbness, and weakness. Schwannomas can also be misdiagnosed as other diseases, particularly neurological diseases. Navarro et al. [20] reported that a 19-year-old male patient with cervical intramedullary schwannoma was initially misdiagnosed as motor neuron disease. Thus, differential diagnosis is very important and should be cautious with schwannomas.

To date, total laminectomy has been regarded as an effective and safe technique in treating intraspinal schwannomas. However, mounting evidence has indicated an increasing rate of approach-related complications, including postoperative spinal instability or progression of spinal deformity [15, 21]. Compared with laminectomy-only, instrumented fixation possesses some evident advantages, especially for dumbbell tumors which are challenging for surgeons [22]. As such, instrumented fixation is imperative after total resection of a large cervical dumbbell schwannoma.

Furthermore, the advantages of instrumented fixation were also indicated by our findings in the current study. Overall, 38 patients undergoing resection of cervical intraspinal schwannomas were incorporated in this study. The patients were divided into two groups based on the different surgical procedures; one was a laminectomy-only group and the other was a laminectomy with instrumented fixation group. The follow-up period is long enough with a mean duration of over 40 months in both groups. Baseline data were well matched between the two groups without a difference. However, only a few patients experienced blood transfusion, and thus, no sufficient data can be compared. The segmental distribution of cervical intraspinal schwannoma was shown similar between the laminectomy-only group and the lateral mass screw group. Postoperatively, pain and neurological impairment significantly improved irrespective of a laminectomy-only group or lateral mass screw group. Seemingly, instrumented fixation did not differ from the laminectomy-only approach in terms of neurological improvement. However, the analyses of postoperative complications suggested that spinal instability and deformity were more likely to exist in the laminectomy-only group as compared with the lateral mass screw group, because the laminectomy-only approach generally leads to the destruction of posterior column structure including posterior ligament complex [15, 16]. We also compared some other postoperative complications including new/worsening sensory symptoms, new/worsening weakness, cerebrospinal fluid leak (4.8%-5.9%), and wound infection, but found no significant differences between the two surgical procedures. The demerits of the lateral mass screw group included longer surgical time due to more operation and higher medical expenses owing to the use of lateral mass screws in comparison with the laminectomy-only group. Some other postoperative complications have been reported in previous studies. Kobayashi et al. [23] reported a case of delayed hydrocephalus which was caused by the leak of cerebrospinal fluid after a cervical schwannoma was resected. Kumar et al. [24] reported that Horner's syndrome happened in the case after a cervical vagal schwannoma was removed.

There are some limitations and shortcomings in this work. First off, this is a retrospective study which might have generated the selection bias. Additionally, this study is a single-center report, not comprehensive enough. At last, this study does not have a large sample size, which could have compromised the power of test and thus is another shortcoming. Therefore, it would be much better if a prospective randomized clinical trial with a large sample size can be performed for further investigation in the future.

## 5. Conclusions

In conclusion, laminectomy with lateral mass screw fixation is an effective and safe approach in treating cervical intraspinal schwannoma, which is likely to be a better choice than laminectomy-only approach, particularly in terms of maintaining postoperative spinal stabilization.

## Figures and Tables

**Table 1:** Demographic data and surgical information (mean ± SD).

Items	Laminectomy-only (*n* = 21)	Lateral mass screw (*n* = 17)	*P* value
Age (yr)	49.4 ± 18.3 (19-72)	47.8 ± 19.1 (17-75)	0.801
Sex	12/9 (M/F)	11/6 (M/F)	0.744^∗^
Duration of symptom	7.3 ± 4.4 months	6.9 ± 4.8 months	0.797
Follow-up (months)	40.6 ± 21.5 (18-67)	43.1 ± 19.8 (20-72)	0.725
Blood loss (ml)	240 ± 180 (105-640)	283 ± 207 (123-955)	0.511
Surgical duration (min)	102.5 ± 31.9 (55-190)	129.7 ± 39.6 (68-220)	0.029
Hospital stay (days)	12.0 ± 4.8 (5-21)	10.5 ± 4.2 (6-18)	0.338
Medical expenses	¥ 14873 ± 3255	¥ 38875 ± 3662	<0.001

^∗^By Pearson chi-square test; the other analyses were determined by independent *t*-tests.

**Table 2:** Comparison regarding VAS score (mean ± SD).

Groups	Pre	PO 3 months	1 year	Last follow-up	*P* value^∗^
Laminectomy-only (*n* = 21)	5.18 ± 2.01	3.15 ± 1.42	1.21 ± 0.78	1.02 ± 0.25	<0.001
Lateral mass screw (*n* = 17)	5.21 ± 2.13	3.11 ± 1.33	1.16 ± 0.85	1.05 ± 0.22	<0.001
*P* value	0.966	0.932	0.856	0.712	—

VAS: visual analogue scale; Pre: preoperation; PO: postoperation. ^∗^Comparison within groups.

**Table 3:** Comparison regarding NDI (mean ± SD).

Groups	Pre	PO 3 months	1 year	Last follow-up	*P* value^∗^
Laminectomy-only (*n* = 21)	24.5 ± 13.8	14.4 ± 11.9	8.2 ± 4.6	4.6 ± 2.5	0.0001
Lateral mass screw (*n* = 17)	23.8 ± 14.6	14.1 ± 12.1	7.2 ± 4.3	4.3 ± 2.4	0.0001
*P* value	0.884	0.941	0.513	0.720	—

NDI: neck disability index (50 points); Pre: preoperation; PO: postoperation. ^∗^Comparison within groups.

**Table 4:** Comparison regarding JOA score (mean ± SD).

Groups	Pre	PO 3 months	1 year	Last follow-up	*P* value^∗^
Laminectomy-only (*n* = 21)	7.1 ± 3.5	10.2 ± 3.2	12.6 ± 2.8	13.0 ± 2.6	<0.001
Lateral mass screw (*n* = 17)	7.3 ± 4.1	10.5 ± 3.4	12.4 ± 3.1	12.8 ± 2.5	<0.001
*P* value	0.876	0.789	0.841	0.819	—

Pre: preoperation; PO: postoperation; JOA: Japanese Orthopedic Association (17 points); ^∗^comparison within groups.

**Table 5:** Summary of postoperative complications.

Complications	Laminectomy-only (*n* = 21)	Lateral mass screw (*n* = 17)	*P* value^∗^
New/worsening sensory symptom	2 (9.5%)	2 (11.8%)	0.758
New/worsening weakness	1 (4.8%)	1 (5.9%)	0.564
CSF leak	1 (4.8%)	1 (5.9%)	0.564
Wound infection	1 (4.8%)	0 (0.0%)	0.915
Spinal instability	6 (28.6%)	0 (0.0%)	<0.001
Spinal deformity	2 (9.5%)	0 (0.0%)	<0.001
Spinal instability/deformity	8 (38.1%)	0 (0.0%)	<0.001

CSF: cerebrospinal fluid. ^∗^By Fisher's exact test.

**Table 6:** Patient satisfaction grades.

Groups	Very satisfied	Satisfied	Dissatisfied	Statistic^∗^
Laminectomy-only (*N* = 21)	15 (71.4%)	4 (19.1%)	2 (9.5%)	*χ* ^2^ = 0.646
Lateral mass screw (*N* = 17)	11 (64.7%)	5 (29.4%)	1 (5.9%)	*P* = 0.724

^∗^Pearson chi-square test between the laminectomy-only group and the lateral mass screw group.

## Data Availability

The data used to support the findings of this study are available from the corresponding author upon request.
